# A liposomal formulation of the synthetic curcumin analog EF24 (Lipo-EF24) inhibits pancreatic cancer progression: towards future combination therapies

**DOI:** 10.1186/s12951-016-0209-6

**Published:** 2016-07-11

**Authors:** Savita Bisht, Martin Schlesinger, Alexander Rupp, Rolf Schubert, Jens Nolting, Jörg Wenzel, Stefan Holdenrieder, Peter Brossart, Gerd Bendas, Georg Feldmann

**Affiliations:** Department of Internal Medicine 3, Center of Integrated Oncology (CIO) Cologne-Bonn, University Hospital of Bonn, Sigmund-Freud-Str. 25, 53127 Bonn, Germany; Pharmaceutical Department, University of Bonn, Bonn, Germany; Institute of Clinical Chemistry and Clinical Pharmacology, University Hospital of Bonn, Bonn, Germany; Department of Pharmaceutical Technology and Biopharmacy, Albert-Ludwigs-Universität, Freiburg im Breisgau, Germany; Department of Dermatology, Center of Integrated Oncology (CIO) Cologne-Bonn, University Hospital of Bonn, Bonn, Germany

**Keywords:** Pancreatic cancer, EF24, Combination therapy, Curcumin analogs, Liposomal nanoparticles, Drug delivery

## Abstract

**Background:**

Pancreatic cancer is one of the most lethal of human malignancies known to date and shows relative insensitivity towards most of the clinically available therapy regimens. 3,5-bis(2-fluorobenzylidene)-4-piperidone (EF24), a novel synthetic curcumin analog, has shown promising in vitro therapeutic efficacy in various human cancer cells, but insufficient water solubility and systemic bioavailability limit its clinical application. Here, we describe nano-encapsulation of EF24 into pegylated liposomes (Lipo-EF24) and evaluation of these particles in preclinical in vitro and in vivo model systems of pancreatic cancer.

**Results:**

Transmission electron microscopy and size distribution studies by dynamic light scattering confirmed intact spherical morphology of the formed liposomes with an average diameter of less than 150 nm. In vitro, treatment with Lipo-EF24 induced growth inhibition and apoptosis in MIAPaCa and Pa03C pancreatic cancer cells as assessed by using cell viability and proliferation assays, replating and soft agar clonogenicity assays as well as western blot analyses. Lipo-EF24 potently suppressed NF-kappaB nuclear translocation by inhibiting phosphorylation and subsequent degradation of its inhibitor I-kappa-B-alpha. In vivo, synergistic tumor growth inhibition was observed in MIAPaCa xenografts when Lipo-EF24 was given in combination with the standard-of-care cytotoxic agent gemcitabine. In line with in vitro observations, western blot analysis revealed decreased phosphorylation of I-kappa-B-alpha in excised Lipo-EF24-treated xenograft tumor tissues.

**Conclusion:**

Due to its promising therapeutic efficacy and favorable toxicity profile Lipo-EF24 might be a promising starting point for development of future combinatorial therapeutic regimens against pancreatic cancer.

**Electronic supplementary material:**

The online version of this article (doi:10.1186/s12951-016-0209-6) contains supplementary material, which is available to authorized users.

## Background

Ductal adenocarcinoma of the pancreas (also referred to as ‘pancreatic cancer’ throughout the following text) remains one of the most dismal of human malignancies to date and accounts for an estimated over 200,000 fatalities every year. Its 5 year overall survival rate of less that 5 % reflects the gloomy prognosis of this disease and is at least in part due to its astonishing resistance to therapeutic intervention [1]. Moreover, as opposed to most other cancer types incident rates are rising and pancreatic cancer has been predicted to become the second most common cause of cancer-related death in the United States within the next decade [2]. Currently available chemotherapeutic regimens that represent the only therapeutic option for the vast majority of locally advanced or metastatic disease states show moderate efficacy and usually extend median overall survival rates by only few weeks [3]. Therefore, the development and rapid, thorough preclinical evaluation of novel therapeutic strategies against pancreatic cancer represents an urgent clinical need.

The plant alkaloid curcumin has long been known in traditional ayurvedic medicine and has recently been shown to exhibit promising antineoplastic activity in pancreatic cancer in vitro and in vivo model systems by our own group as well as by others [4–7]. The synthetic curcumin monoketone derivative 3,5-bis(2-fluorobenzylidene)-4-piperidone (EF24) shows potent in vitro anticancer activity [8], however, similar to the parent compound curcumin, further clinical development into a suitable drug candidate is hampered by its poor water solubility and bioavailability.

To overcome these difficulties, this study reports creation and preclinical evaluation of a liposomal nanoformulation of EF24 (Lipo-EF24). Liposomal drug encapsulation is now well established, and in fact liposomal nanoformulations of cytotoxic chemotherapeutics are routinely used in clinical oncology by now. A prominent example is the use of liposomal doxorubicin formulations (e.g. Caelyx, Myocet) instead of free drug, which showed enhanced therapeutic efficacy and considerably better toxicity profiles, particularly far lesser cardiotoxicity [9, 10]. Therefore, further translational development of Lipo-EF24 as presented here into clinical evaluation appears to be feasible in principle.

The liposomal nanoformulation of EF24 generated here (designated “Lipo-EF24”) showed potent in vitro and in vivo antineoplastic activity in clinically relevant pancreatic cancer models. Moreover, a very favorable toxicity profile was observed, rendering Lipo-EF24 a promising candidate for further development and a potential partner for future experimental combination regimens.

## Methods

### Chemicals

1-Palmitoyl-2-oleoyl-sn-glycero-3-phosphocholine (POPC) and 1,2-distearoyl-sn-glycero-3-phosphoethanolamine-*N*-[poly(ethylene glycol)-2000] (PEG-DSPE) were purchased from Avanti Polar Lipids (Alabaster, AL, USA). Cholesterol, EF24 (>99 % purity) and curcumin were purchased from Sigma-Aldrich (Steinheim, Germany). Gemcitabine was obtained from NetQem LLC (Durham, NC, USA) and dissolved in sterile NaCl solution (0.9 % w/v) on the day of use.

### Synthesis of void and EF24-loaded liposomes

Liposomes were prepared using the film hydration method. Briefly, PEG-stabilized void liposomes were prepared by a mixture of POPC, cholesterol, and PEG-DSPE, at a molar ratio of 65:30:5 mol%. EF24 containing liposomes were produced at molar ratio of 60 mol% POPC, 30 mol% Cholesterol, 5 mol% PEG-DSPE, and 5 mol% EF24. Unilamellar liposomes were obtained by extruding multilamellar vesicles through polycarbonate membranes as previously described [11]. Briefly, the mixture of phospholipids and EF24 in chloroform, respectively, was dried in a rotary evaporator under reduced pressure. The resulted lipid film was hydrated with phosphate buffered saline (PBS) to reach a final lipid concentration of 50 µmol/mL. The multilamellar vesicles, obtained by thoroughly mixing the aqueous solutions of lipids, were extruded five times through a 200 nm and five times through a 100 nm polycarbonate membrane (Isopore™, Millipore, Schwalbach, Germany) at 65 °C using a Lipex™ 10 mL Thermobarrel Extruder (Lipex™ Biomembranes, Inc., Vancouver, Canada). EF24 excess was removed by gel permeation chromatography (Sephadex G-50^®^; Sigma-Aldrich, Steinheim, Germany). Phospholipid concentration was quantified with a standard phosphate assay [12–14].

Incorporation of EF24 in liposomes was determined by gas chromatography–mass spectrometry (Hewlett Packard 5890, Series II, Quadrupol MS, column CP-Sil m8, 50 m, 0.25 µm × 0.25 mm, Böblingen, Germany).

### Dynamic light scattering (DLS) measurements

The average size and the size distribution of the formed liposomes were measured using a Zetatrac system (Microtrac Europe GmbH, Meerbusch, Germany). Briefly before the measurements, the liposomes were diluted in PBS and filtered through Millipore filters with an average pore size of 0.22 µm. The measurements were performed at room temperature and at a scattering angle of 180°. All measurements were done in triplicates and mean sizes were calculated.

### Transmission electron microscopy (TEM)

TEM pictures of void and EF24-loaded liposomes were acquired using a Leo 912 OMEGA instrument (Carl Zeiss, Oberkochen, Germany) operating at 120 kV. Briefly, a drop of diluted solution of liposomes was coated on carbon-coated copper grids (Quantifoil Micro Tools GmbH, Jena, Germany) and was then immediately shock-frozen in liquid ethane before loading in the microscope. For each probe three grids were prepared and all digital images were captured at a magnification of 6–12K using a Proscan HSC 2 camera [15].

### Cell culture

Human pancreatic cancer cell lines were cultured in Dulbecco’s Modified Eagles Medium (DMEM, PAA Laboratories, Pasching, Austria) supplemented with 10 % FBS, 0.1 mM non-essential amino acids solution, 1 mM sodium pyruvate, and 1 % penicillin/streptomycin (all PAA Laboratories, Pasching, Austria) as well as 5 μg/mL plasmocin (InvivoGen, San Diego, CA). Immortalized non-malignant human pancreatic epithelial cells (hTERT-HPNE) were cultured as described elsewhere [16, 17]. All cell lines were grown in a humidified atmosphere at 37 °C in the presence of 5 % CO_2_ and were regularly checked for mycoplasma infection using a PCR based assay as previously described [18].

### Cell viability assays

Cell viability was determined using 3-(4,5-dimethyl-2-yl)-5-(3-carboxymethoxyphenyl)-2-(4-sulfophenyl)-2H-tetrazolium (MTS) assays as previously described [4, 19]. Briefly, 2000 cells per well were plated in full growth media and treated with either free EF24 or liposome-encapsulated EF24 for 72 h, respectively. At 72 h, the assay was terminated and relative growth inhibition compared to mock treated cells was determined using CellTiter 96 reagent (Promega, Madison, WI), as described in the manufacturer’s protocol. All experiments were set up in triplicates.

### Proliferation assay (cell trace violet dye staining)

Cell proliferation was measured in vitro using Cell Trace Violet Cell Proliferation Kit (ThermoFisher Scientific GmBH, Schwerte, Germany). In brief, 1 × 10^6^ cells were labeled with a vital dye cell trace violet (5 µmol/L) according to manufacturer’s protocol, seeded in 12 well plates and later treated with void liposomes as well as EF24 loaded liposomes at 5 and 10 µM respectively. After 48 h of treatment, cells were harvested, washed and analyzed for violet dye fluorescence using flow cytometry.

### Clonogenicity assays

Cells were seeded in six-well plates at a density of 1000 cells per well in the presence of different concentrations of void or EF24-containing liposomes, respectively. After 24 h, medium containing liposomal solutions was replaced with normal medium and colonies were allowed to grow. Once colonies became visible, cells were fixed and stained using 0.05 % crystal violet (Sigma Aldrich, Steinheim, Germany). Colonies were counted and colony counts were normalized to the mean colony count of void liposome-treated cells.

### Anchorage independent growth assays

Soft agar assays were set up in six well plates as described previously [17]. In brief, a base agar layer was formed by mixing 2 mL of media and 1 % agarose containing different concentrations of either void liposomes or liposomes encapsulating EF24, respectively. Next, on top of it a second layer of 2 mL media containing 0.7 % agarose and 10,000 cells were poured in the presence of void or EF24-loaded liposomes and allowed to solidify. Finally, 2 mL of media was added on top of the agarose layers and the plates were incubated at 37 °C for 2 weeks. After 2 weeks, the visible colonies were stained and visualized using trans-UV illumination (BioRad, Hercules, CA). Colonies were counted and colony counts were normalized to the mean colony count of the respective void liposomes controls. All assays were set up in triplicates.

### Western blot analyses

Cells were lysed using radioimmunoprecipitation assay buffer (RIPA: 1 % IgepalCA630, 0.5 % sodium deoxycholate, 0.1 % SDS, 2 mM EDTA) supplemented with protease and phosphatase inhibitor cocktails (Sigma Aldrich, Steinheim, Germany). 50 µg of total protein was separated on 4–12 % Nupage bis–tris gels (Life Technologies, Darmstadt, Germany) and transferred onto PVDF membranes (Millipore, Billerica, MA, USA). The blots were blocked using either 5 % (w/v) BSA or 5 % (w/v) milk in TBST for 1 h and then probed using primary antibodies against the p65 subunit of Nuclear Factor kappaB (NF-kappaB), Inhibitory Protein I-kappa-B-alpha, Caspase-3, PARP or GAPDH (1:1000, Cell Signaling, Danvers, MA) as well as HRP-coupled secondary antibodies directed against rabbit or mouse IgG, respectively (1:2000; Cell Signaling, Danvers, MA). Detection was performed as previously described [20]. Cytoplasmic and nuclear proteins were extracted using the NE-PER nuclear and cytoplasmic extraction kit (Thermo-Fischer Scientific, Schwerte, Germany).

### Ex vivo red blood cell (RBC) hemolysis assay

Fresh mouse blood was collected (n = 6) and the red blood cells (RBCs) were separated from the serum by centrifugation. The collected blood cells were then washed twice with PBS and resuspended in PBS (pH 7.4). The RBC suspension (2 % v/v) was then incubated with varying concentrations of void or EF24-loaded liposomes at 37 °C for 1 h. After incubation, the supernatant was centrifuged at 500×*g* for 5 min and subjected to measurement for hemoglobin release by microplate reader (FLUOstar Optima, BMG Labtech, Offenburg, Germany) at 450 nm. Samples incubated with 1 % Triton X-100 served as positive controls (100 % hemolysis), addition of PBS served as negative controls (0 % hemolysis), respectively.

### Sample preparation for LC–MS/MS measurements

LC–MS/MS samples were prepared by adding 4 µL of serum to a mixture of 96 µL methanol, 96 µL H_2_O and 4 µL of ISTD stock solution (=1 mg/L ISTD in methanol). After thorough mixing, samples were transferred into MS vials and stored in the sample manager at 10 °C. All solvents were of LCMS grade.

### Pharmacokinetic analysis of EF24 loaded Liposomes using LC–MS/MS

For pharmacokinetic analyses, a cohort of 24 mice were administered a single dose of liposomal EF24 (10 mg/kg) intravenously through tail vein. Serum samples (2–4 mice per time point) were collected at 0.25, 1, 2, 3, 4, 6, 8 and 24 h and stored at −80 °C prior to and after use. LC–MS/MS analyses were carried out on a Waters Xevo TQ-S Triple-quad system equipped with a Waters Acquity I-Class UPLC system, an FTN sample manager, binary solvent manager and TUV detector. A Waters Acquity UPLC BEH C18 column (130 Å, 1.7 µm, 2.1 × 100 mm) together with a Waters Acquity UPLC BEH C18 VanGuard pre-column (130 Å, 1.7 µm, 2.1 × 5 mm) was used for separation. Data recording was achieved with the MassLynx 4.1 software package, data processing and quantification was performed with the integrated TargetLynx software using a quadratic regression model. Detailed LC–MS/MS methodology and data quantification is provided as Additional file 1.

### Generation of xenografts and drug treatment

Animal experiments described comply with Directive 2010/63/EU and were approved by the government of the state of North Rhine-Westphalia (AZ84-02.04.2011.A138). Mice were maintained according to the guidelines of the Federation of European Laboratory Animal Science Associations (FELASA). Subcutaneous xenografts were generated by injecting 1 × 10^6^ MIAPaCa cells suspended in a total volume of 200 µL [PBS/Matrigel (BD Biosciences), 1:1 (v/v), prechilled to 4 °C] into 5–6 weeks old athymic nu/nu mice (Jackson Laboratory, Maine, USA). After 2 weeks, subcutaneous tumor volumes were measured using digital calipers (Milomex, Pulloxhill, UK) and calculated using the formula V = 1/2(ab^2^), where a is the longest and b is the shortest orthogonal tumor diameter [21]. Mice were then randomized and divided into four cohorts of eight animals each and administered one of the following regimens: (a) void liposomes, (b) EF24 loaded in liposomes at a dose of 10 mg/kg i.v. on alternate days, (c) gemcitabine at a dose of 20 mg/kg i.p. twice weekly, or (d) combination of EF24-loaded liposomes and gemcitabine. Tumor volumes and body weights were measured once weekly. After 3 weeks, tumors and visceral organs were harvested and preserved in 10 % neutral buffered formalin or snap-frozen for further analyses.

### Statistical analysis

Two-tailed Student’s t test and Mann–Whitney U test were performed using Graph Pad Prism for Windows version 6. Kruskal–Wallis analyses were done using SPSS for Microsoft Windows. p < 0.05 was regarded as statistically significant. Unless indicated otherwise, results are shown as mean ± SD. Further analyses are described in the Additional file 1.

## Results

### Synthetic analog of curcumin EF24 shows more potent growth inhibition in pancreatic cancer cell lines in vitro

**Fig. 1:**
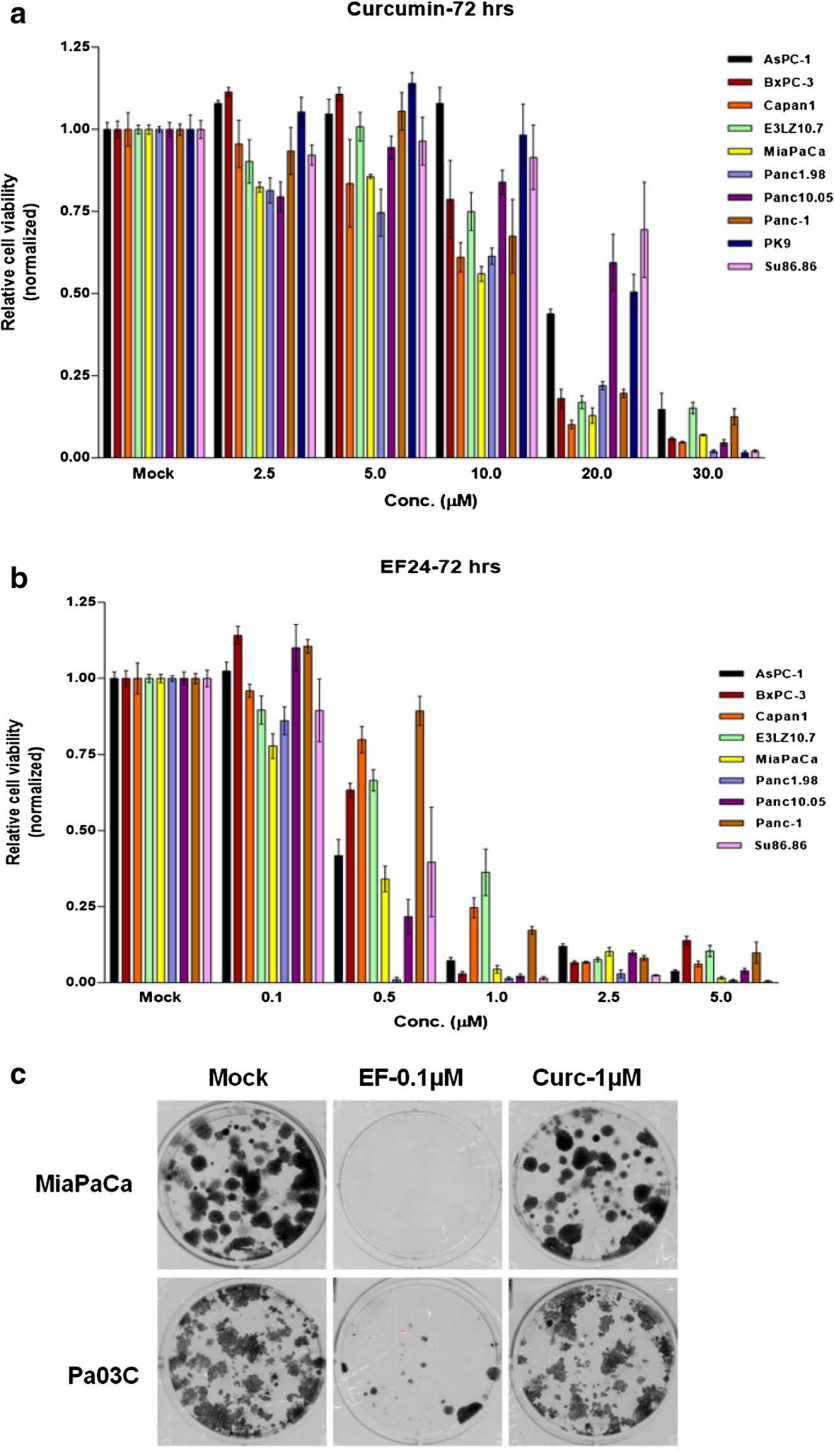
Growth inhibition of pancreatic cancer cell lines by EF24 and by curcumin. A panel of ten pancreatic cancer cell lines was exposed to increasing doses of **a** curcumin or **b** EF24 for 72 h, and cell viability was determined using MTS assays. Across all cell lines, EF24 inhibited viability at approximately 10- to 20-fold lower IC_50_ as compared to curcumin. **c** Similar effects were observed in clonogenic assays, where EF24 abrogated the ability of pancreatic cancer cells to form colonies at concentrations that were about tenfold lower than the required curcumin doses (all experiments were done in triplicates and repeated at least once; the figure shows pictures from two representative experiments)

The therapeutic activity of EF24 was tested and compared to its parent compound curcumin in a panel of ten different pancreatic cancer cell lines using MTS assays (Fig. 1a, b). As shown in the figure, EF24 inhibited net cell growth of pancreatic cancer cells in a dose dependent manner and with almost 10- to 20-fold lower IC50 as compared to curcumin across various cell lines. Moreover, EF24 also abrogated the ability of pancreatic cancer cells to form colonies at about 10-fold lower concentrations than curcumin in two different cell lines, MiaPaCa and Pa03C, respectively (Fig. 1c). Thus, our results show enhanced antineoplastic activity of EF24 on pancreatic cancer cell lines as compared to curcumin, which is in line with previous studies in other cancers [22–24].

### Synthesis and characterization of pegylated void and liposomal EF24

**Fig. 2:**
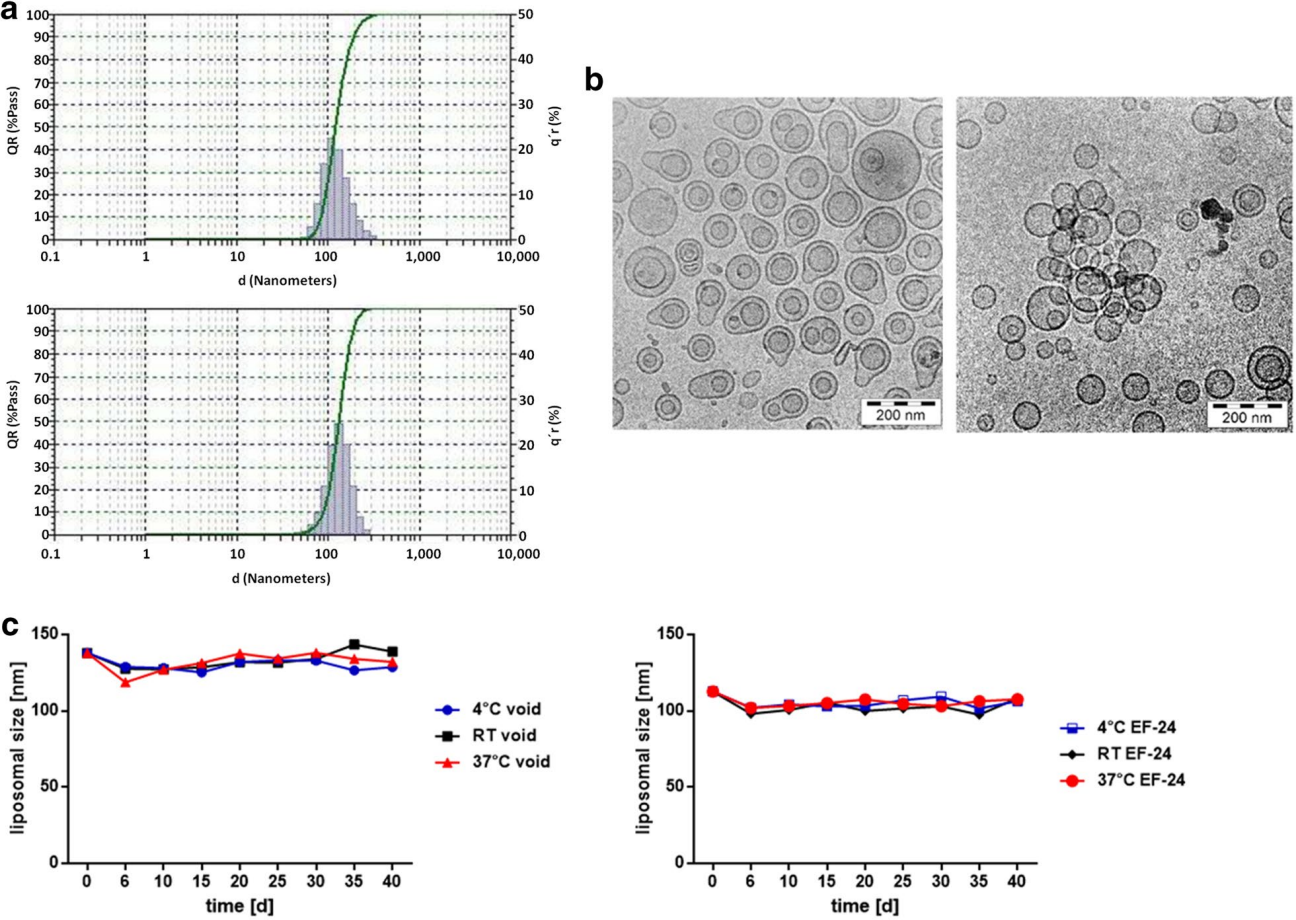
Synthesis and characterization of void and EF24-containing PEGylated liposomes. Pegylated liposomes synthesized using a lipid hydration method were further characterized using DLS and TEM. **a** DLS of void and EF24-loaded liposomes revealed a narrow size distribution with an average diameter of less than 150 nm. **b** Transmission electron microscopy of void (*left panel*) and EF24-containing liposomes (*right panel*) demonstrated spherical morphology and an average diameter of around 120 nm, in line with the data obtained by DLS. **c** The stabilities of void and EF24-loaded liposomes were determined at three different temperatures (4, 20 and 37 °C) using DLS over a period of 40 days

Hydration technique was used to generate liposomes in the presence of EF24, followed by an extrusion procedure to obtain a homogenous particle size. Due to its lipophilic structure, EF24 was predominantly encapsulated in the interior of liposomal bilayers. The average diameter and size distribution of void and EF24-loaded liposomes were routinely measured using dynamic light scattering (DLS) as illustrated in Fig. 2a. Both void liposomes as well as liposomes loaded with EF24 showed a narrow size distribution with an average diameter of less than 150 nm. Likewise, transmission electron microscopy (TEM) of the obtained liposomal particles demonstrated intact, round vesicles with an average diameter of <150 nm, and no difference in mean size was observed between void and EF24-loaded liposomes. Moreover, mixing of phospholipids and EF24 at different ratios revealed a maximum encapsulation of 5 mol% of EF24 in liposomes without affecting the structural integrity of liposomes (Fig. 2b). Loading of EF24 in liposomes was determined using gel permeation chromatography and unencapsulated EF24 in the eluted buffer was quantified by means of gas chromatography–mass spectrometry (GC–MS). No EF24 was detected in different eluates thereby confirming complete and stable incorporation of EF24 within liposomal phospholipid bilayers. Next, liposomal particles thus prepared were tested for their stability and shelf life at different storage temperatures (4, 20, and 37 °C, respectively) using DLS. Of note, liposomes displayed high stability and showed no agglomeration or change in their average diameter over 40 days (Fig. 2c), possibly due to PEGylation of the liposomal surface.

### Pegylated liposomal EF24 (Lipo-EF24) impairs growth and abrogates colony formation of pancreatic cancer cell lines in vitro

**Fig. 3:**
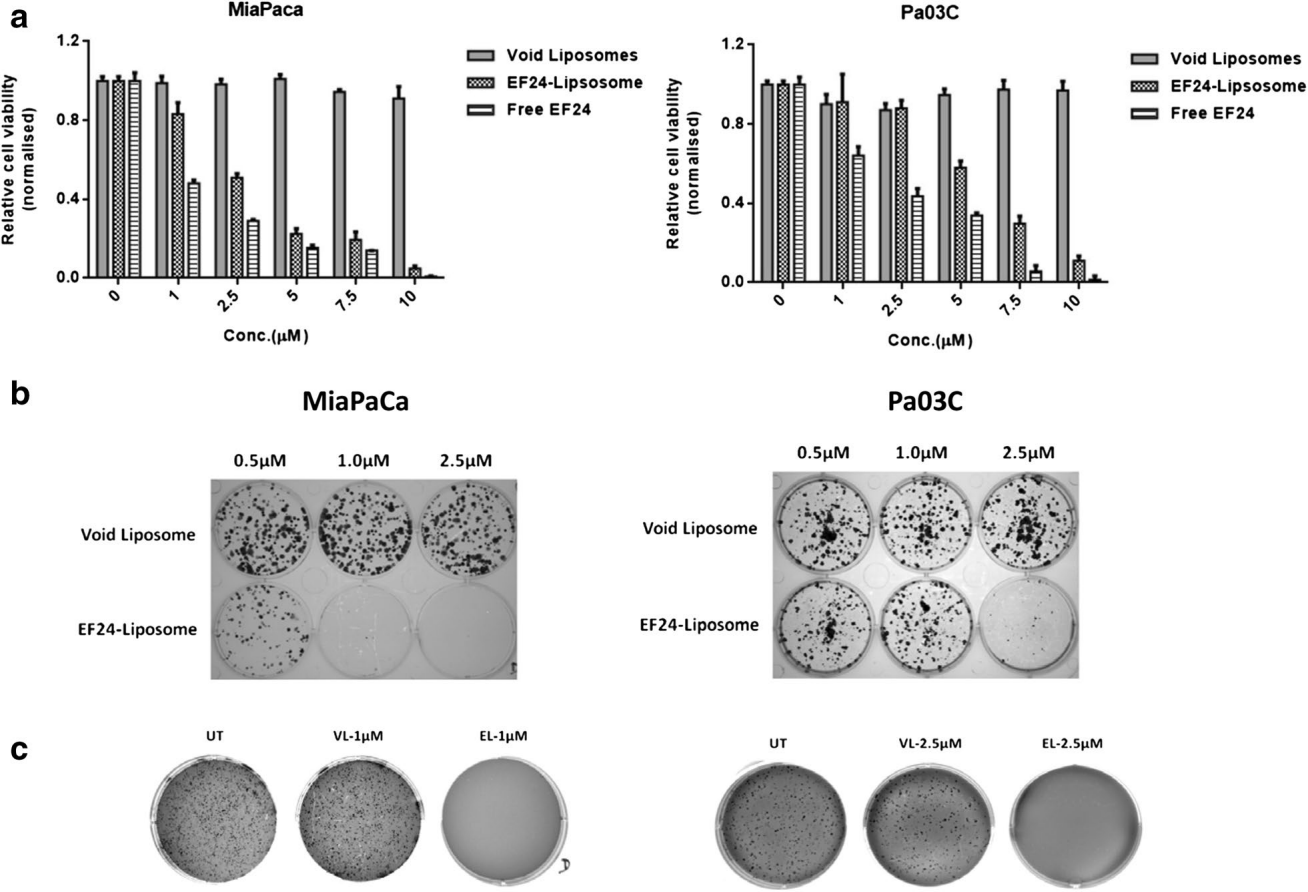
Lipo-EF24 inhibits growth and clonogenicity of pancreatic cancer cell lines. **a** MTS assays were performed using equivalent doses of free and liposomal EF24 in two different pancreatic cancer cell lines, MIAPaCa and Pa03C. In both cell lines, liposomal EF24 demonstrated marked growth suppression comparable to that of free drug. Further, liposomal EF24 caused significant, dose-dependent reduction in the ability of these cells to form colonies in replating assays (**b**) and inhibited anchorage-independent growth in soft agar (**c**). Void liposomes showed no significant effect in any of these cell lines. Representative images from one of three independent experiments are shown

**Fig. 4:**
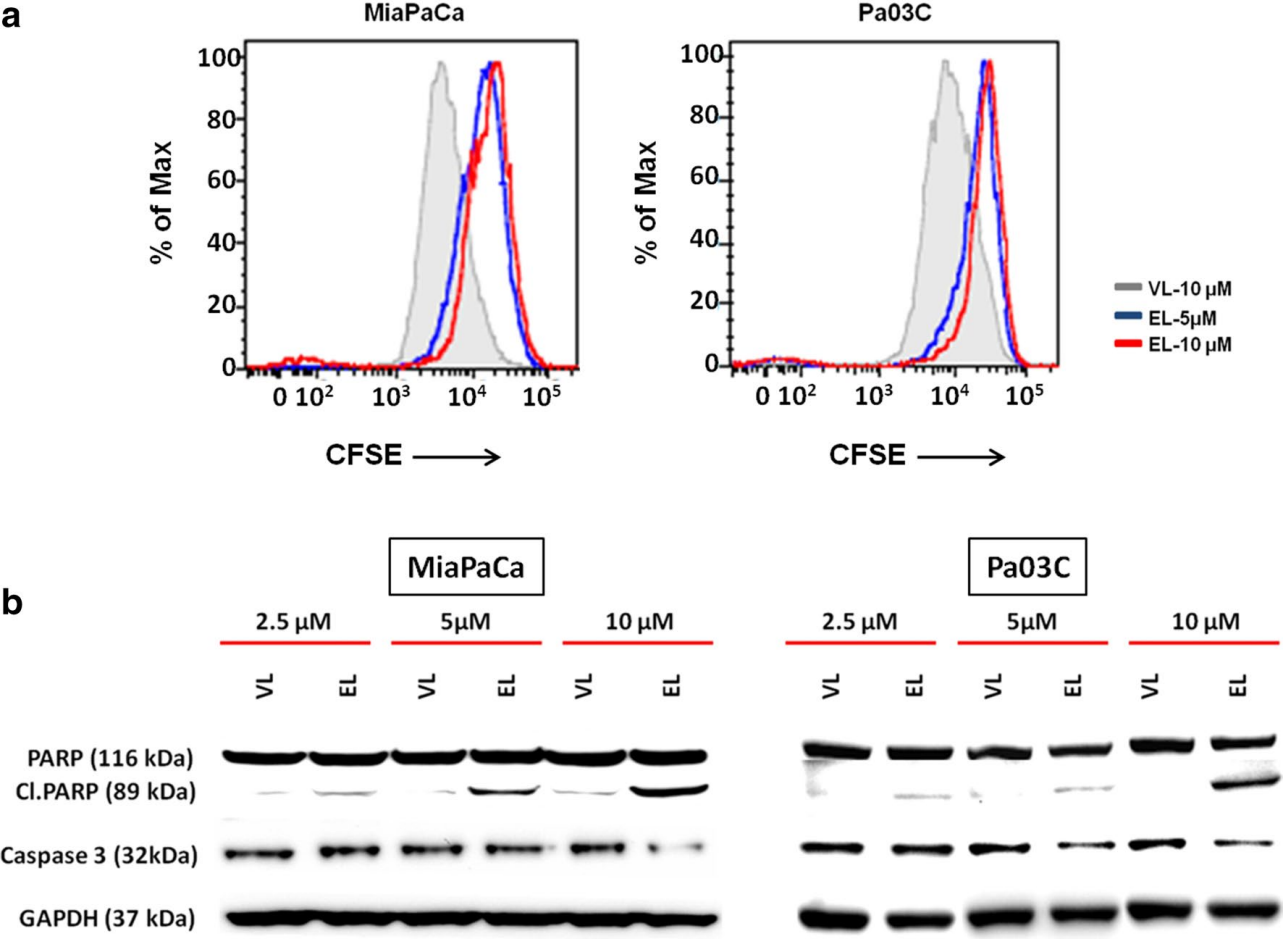
Lipo-EF24 inhibits proliferation and induces apoptosis in pancreatic cancer cells. **a** CFSE staining assays were used to measure the effect of Lipo-EF24 on pancreatic cancer cell proliferation. CFSE-labeled MIAPaCa and Pa03C cells were treated with Lipo-EF24 at concentrations of 5 and 10 µM or void liposomes for 48 h and then analysed using flow cytometry. Proliferation was indicated by a decrease of fluorescence intensity. **b** In order to determine apoptosis rates, MIAPaCa and Pa03C cells were treated with indicated concentrations of void liposomes or Lipo-EF24 for 24 h and protein expression levels of cleaved PARP or procaspase-3 were quantified from whole cell lysates by immunoblotting

The therapeutic efficacy of liposomal EF24 was examined in two different human pancreatic cancer lines (MIAPaCa and Pa03C) and directly compared to free EF24 or void liposomes, respectively, using MTS assay. As shown in Fig. 3a, Lipo-EF24 significantly inhibited growth of both cell lines in a dose-dependent manner and to an extent that was comparable to that of free EF24 while void liposomes showed negligible effects on cell growth. Moreover, when assessed using replating assays, both the cell lines mentioned above failed to grow colonies from single cell suspensions in the presence of liposomal EF24 but readily formed colonies when exposed to void liposomes (Fig. 3b). Likewise, liposomal EF24 potently and reproducibly abrogated anchorage independent growth of MIAPaCa and Pa03C cells in softagar, while such an effect was not observed for void liposomes or untreated controls (Fig. 3c). Next, the effect of liposomal EF24 on cellular proliferation was analyzed using a CFSE dilution assay, which relies on the depletion of fluorescence intensity of CFSE with cell division. Of note, addition of Lipo-EF24 to culture media at concentrations of 5 or 10 µM on CFSE labeled cells for 48 h significantly decreased proliferation of both cell lines as compared to controls (Fig. 4a).

### Lipo-EF24 induces apoptosis in pancreatic cancer cells

Previous studies suggested that EF24 might induce apoptosis in various different cancers including ovarian, gastrointestinal and breast cancer [23, 25, 26]. Therefore, in order to test whether EF24 retains its biological activity despite being encapsulated into liposomal particles, MIAPaCa or Pa03C pancreatic cancer cells were treated with different concentrations of either void liposomes or Lipo-EF24 for 24 h and expression levels of cleaved poly (ADP-ribose) polymerase (cl. PARP) as well as caspase-3 were determined by western blot analysis. As demonstrated in Fig. 4b, Lipo-EF24 induced cleavage of PARP in both cell lines tested in a dose-dependent manner. Expression levels of caspase-3 were found to be reduced by Lipo-EF24 at higher concentrations of 10 µM in MIAPaCa, or at 5 and 10 µM in Pa03C cells, respectively. Void liposomes on the other hand did not alter expression patterns in either of these cell lines.

### Lipo-EF24 suppresses NF-kappaB activation by inhibiting phosphorylation and degradation of I-kappa-B-alpha

**Fig. 5:**
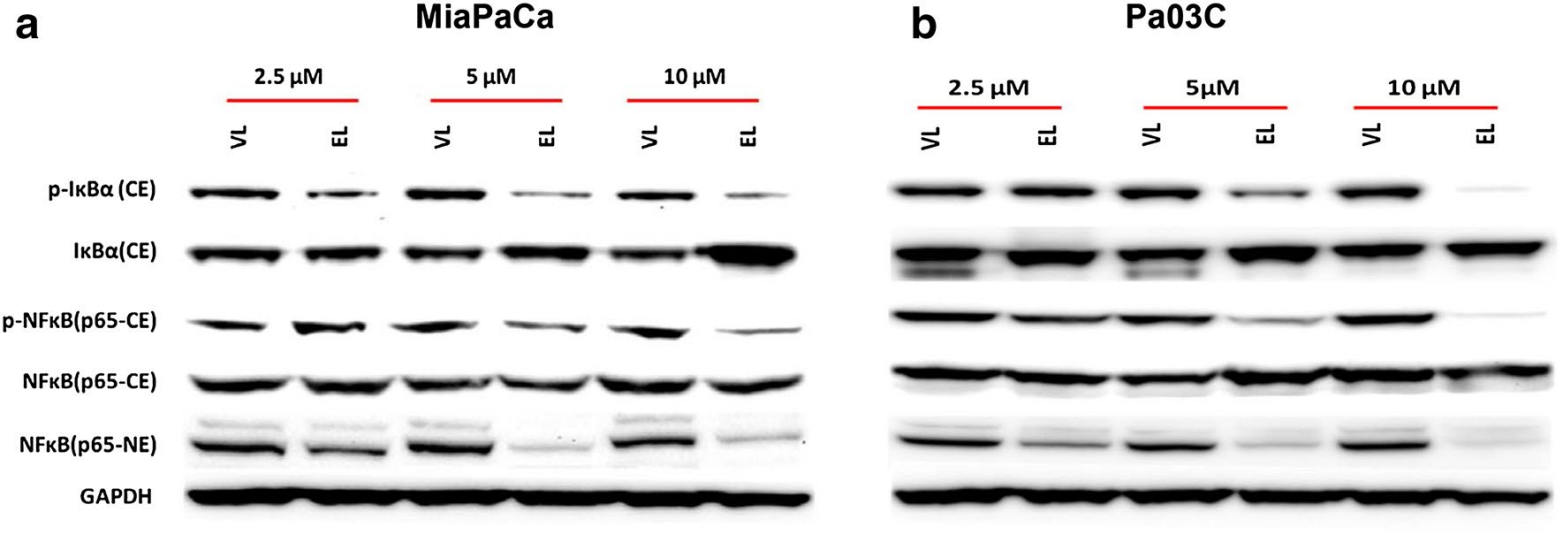
Lipo-EF24 blocks I-kappa-B-alpha (IkBa) phosphorylation and NF-kappaB activation. The effect of void liposomes (VL) or Lipo-EF24 (EL) on NF-kappaB pathway activity in **a** MIAPaCa or **b** Pa03C cells was assessed by quantifying the protein expression levels of phospho-I-kappa-B-alpha (p-IκBα), I-kappa-B-alpha (IκBα), phospho-NF-kappaB-p65 (p-NFκB p65) and NF-kappaBp-65 (NFκB p65) in cytoplasmic (CE) as well as nuclear (NE) extracts, GAPDH was used as loading control

The oncogenic NF-kappaB pathway is constitutively active in human pancreatic cancer [27], moreover curcumin has previously been identified as potent inhibitor of NF-kappaB signaling [28]. A recent study suggests that the curcumin derivative EF24 suppresses NF-kappaB activation by directly inhibiting the degradation of I-kappa-B-alpha [24], the inhibitory cytosolic subunit of NF-kappaB, whose phosphorylation and subsequent degradation is a prerequisite for activation of the NF-kappaB pathway. Hence, in order to measure the effects of Lipo-EF24 on NF-kappaB signaling, cytoplasmic and nuclear proteins were extracted from treated MIAPaCa and Pa03C cells, respectively, and subjected to western blot analysis. As shown in Fig. 5, liposomal EF24 effectively inhibited phosphorylation of I-kappa-B-alpha as well as NF-kappaB-p65 and subsequent nuclear translocation of NF-kappaB-p65 in a dose-dependent manner. Moreover, a marked increase in the expression levels of total I-kappa-B-alpha was also observed in MIAPaCa cells treated with Lipo-EF24 at a concentration of 10 µM, indicating that EF24 protects I-kappa-B-alpha from subsequent cytosolic degradation.

### Inertness of void liposomes in vitro and in vivo

**Fig. 6:**
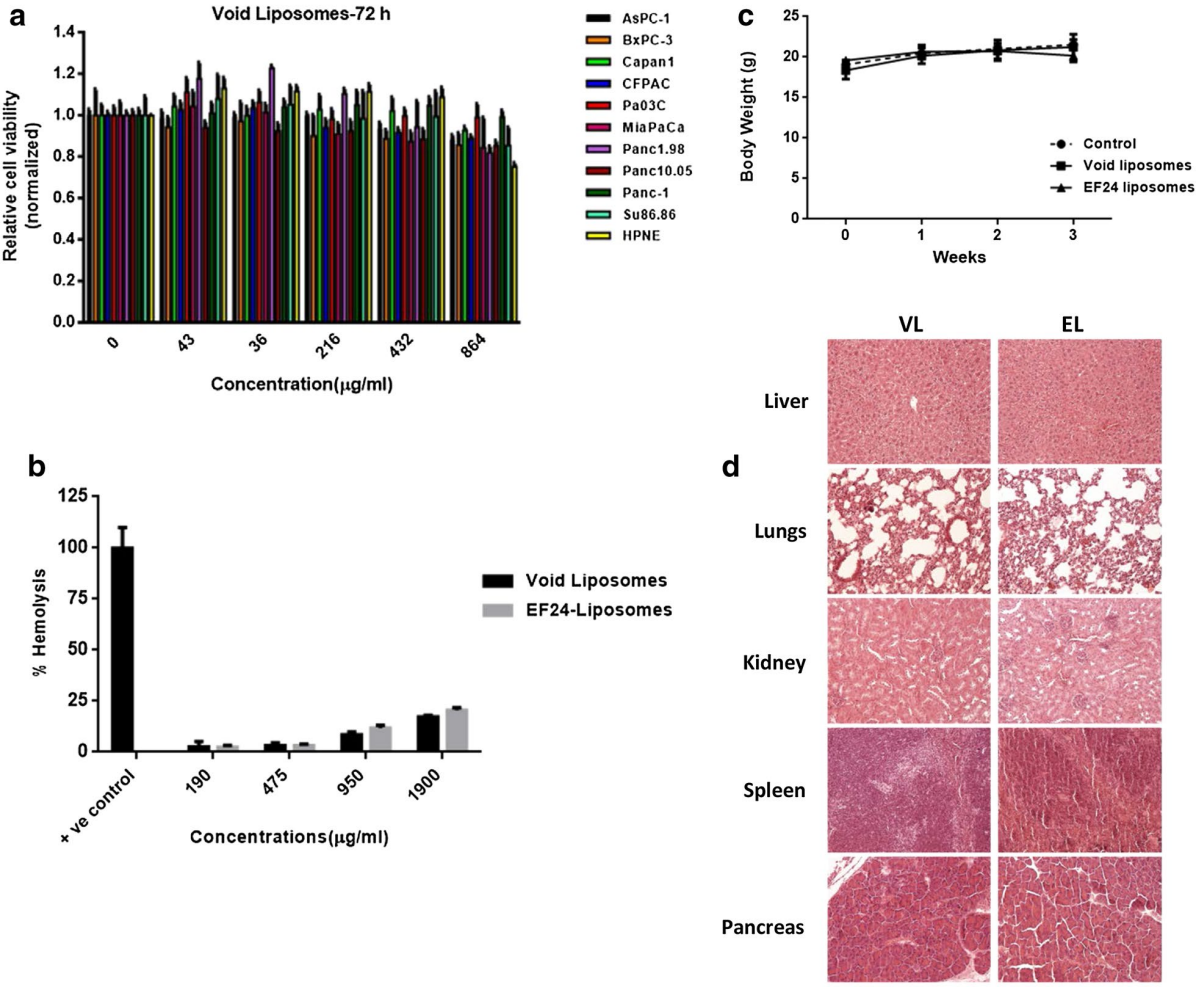
Toxicity profile of void liposomes. **a** The toxicity of void liposomes was examined in ten pancreatic cancer cell lines as well as non-malignant HPNE cells. These cells were exposed to increasing doses of void liposomes for 72 h and growth inhibition was determined using MTS assays. **b** The hemolytic activity of void or EF24-loaded liposomes on erythrocytes (RBCs) was evaluated using ex vivo RBC hemolysis assays. **c** In vivo toxicity studies were performed by systemic administration of void liposomes, Lipo-EF24 (both at 10 mg/kg i.v. thrice weekly for a total of 3 weeks) or PBS as mock treatment control to CD1 wildtype mice. Body weights in the three respective treatment arms were assessed on a weekly basis and no significant differences in mean body weights were observed during the course of experiment. **d** Histopathological assessment of major organs obtained from these mice did not show any discernible abnormalities or other evidence of toxicity. Representative HE sections of major organ sites obtained from mice treated with void liposomes (VL) or Lipo-EF24 (EL) are shown

Cytotoxicity of pegylated void liposomes was assessed in an extended panel of ten pancreatic cancer cell lines and one immortalized benign human pancreatic ductal epithelial cell line (HPNE). Void liposomes did not show any cytotoxic effect over a wide dose range from 43 µg/mL up to 864 µg/mL (Fig. 6a). Next, ex vivo hemolysis assays were performed to check for possible adverse effects of liposomes on survival of red blood cells. For this, separated murine red blood cells were exposed to void or EF24-loaded liposomes, respectively, at concentrations ranging from 190 to 1900 µg/mL. Even at the highest concentrations tested (i.e. nearly 2 mg/mL), the hemolytic activity of void or EF24-loaded liposomes measured did not exceed 20 % as compared to positive control samples (100 % hemolysis) (Fig. 6b). To assess for in vivo toxicity, void or EF24-loaded liposomes, respectively, were administered to CD1 wildtype mice at a dose of 10 mg/kg by i.v. injection thrice weekly for 3 weeks, mock injections of PBS served as controls. Of note, mice treated with either void or drug-loaded liposomes did not show any signs of distress, body weight loss or any behavioral conspicuousness as compared to mock treated animals during the entire course of the experiment (Fig. 6c). Thorough necropsy and examination of major organs, including liver, lungs, kidney, spleen, pancreas, did not reveal any discernible gross or histomorphological abnormalities (Fig. 6d).

### In vivo pharmacokinetic analysis of Lipo-EF24 using liquid chromatography–mass spectrometry (LC–MS/MS)

**Fig. 7:**
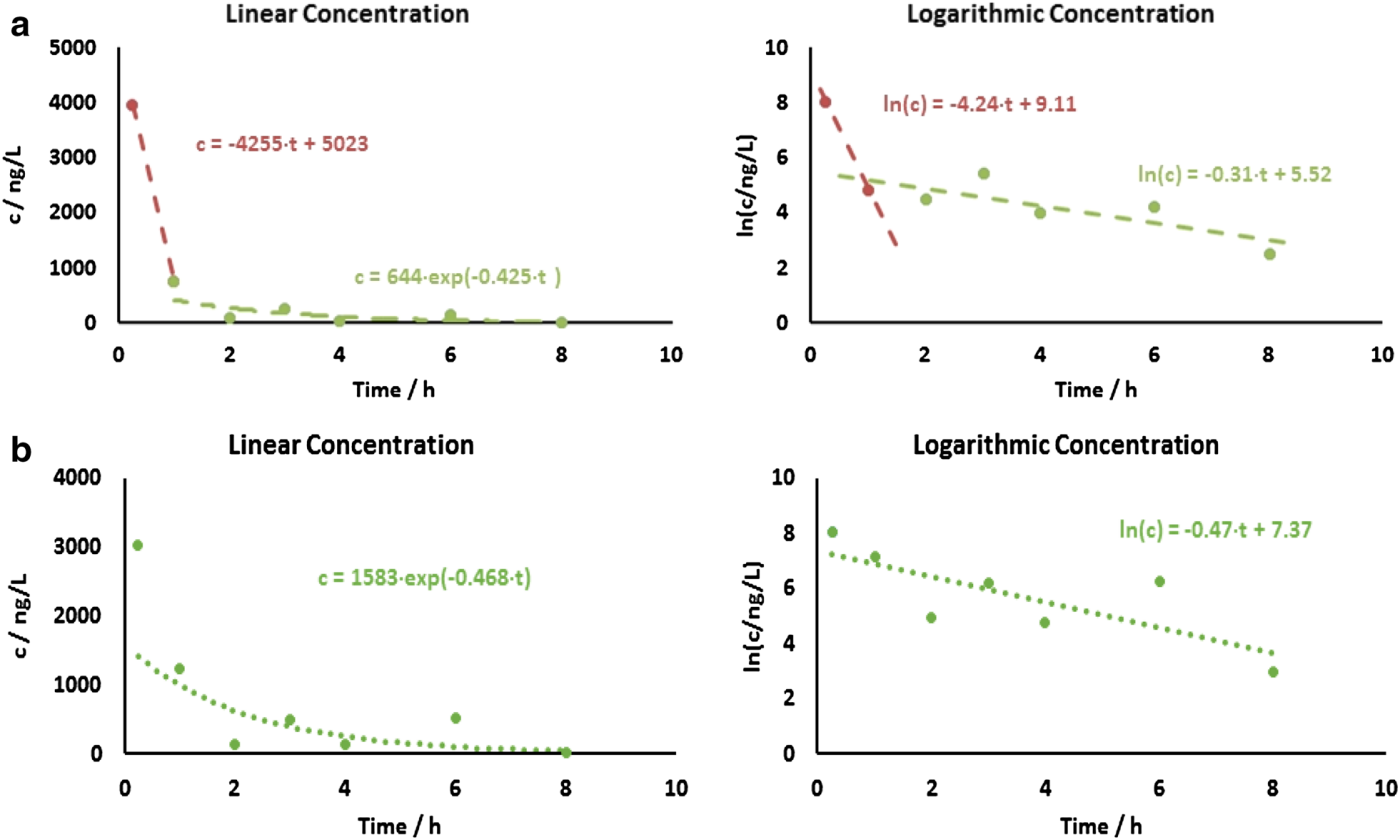
In vivo pharmacokinetics of EF24 loaded liposomes. **a** Graphical representation of the results from LC–MS/MS measurements. *Left* linear scale; *right* semilogarithmic scale. Plasma concentrations of EF24 were determined at 0.25, 1, 2, 3, 4, 6, 8 and 24 h after intravenous administration of liposomal EF24 formulation, given as single dose equivalent to 10 mg/kg EF24. For each time point data from at least two to four mice were analysed. **b** Graphical representation of the results of free (not encapsulated) EF24 solubilized in DMSO from LC–MS/MS measurements. *Left* linear scale; *right* semilogarithmic scale

The mice were injected intravenously with a single dose of encapsulated EF24 (10 mg/kg) at t = 0 h. Peripheral blood samples drawn at different time intervals (0.25, 1, 2, 3, 4, 6, 8 and 24 h, respectively) were quantified by means of LC–MS/MS and the results obtained were averaged afterwards. The data thus acquired were plotted on a linear and semi-logarithmic scale (Fig. 7). As evident from these graphs, a rapid initial decrease in mean EF24 plasma concentrations within the first hour was followed by a subsequent phase of less pronounced decline between t = 1 h and t = 8 h. At 24 h, mean plasma levels of EF24 were found to be below the detection limit. Thus, a distribution phase within the first hours and an elimination phase (after t = 1 h) could be differentiated. Both phases were fitted separately on a semi-logarithmic scale, which yielded rate constants of k_dist_ = 4.25 h^−1^ and k_elim_ = 0.31 h^−1^, respectively. Corresponding half-life values were t_1/2,dist_ = 0.16 h and t_1/2,elim_ = 2.23 h. Due to sharp initial concentration decrease, the area under the concentration–time curve (AUC) was determined by both linear and logarithmic trapezoidal method, which yielded corresponding values of AUC_lin_ = 3074 ng h L^−1^ and AUC_log_ = 2456 ng h L^−1^, respectively. Upon extrapolation from t = 0.25 h to t = 0 h, values amount to AUC_lin,0_ = 4197 ng h L^−1^ and AUC_log,0_ = 3573 ng h L^−1^, respectively. Additionally, extrapolating the concentration c to t = 0 h results in c_max_ = 5023 ng L^−1^ (Fig. 7a). For comparison, the pharmacokinetic profile of pure EF24 solubilized in DMSO was also determined by LC–MS/MS in the time regime between 0.25 and 8 h after intravenous injection of a single dose of EF24 (10 mg kg^−1^). Without the liposomic encapsulation EF24 shows no sign of a distribution phase, but is constantly eliminated with a rate constant of 0.4683 h^−1^ and a half-life of t_1/2_ = 2.14 h. The measured peak concentration at t = 0.25 h is roughly 75 % of the peak concentration of encapsulated EF24. Extrapolating the first three concentration time points to t = 0 yields an initial concentration of roughly 5500 ng L^−1^, which is slightly higher than in the case of EF24 encapsulated in liposomes. Accordingly, the AUC also amounts to a higher value of 5258 ng L^−1^ h^−1^ (Fig. 7b).

### Lipo-EF24 shows synergistic in vivo growth inhibition in combination with gemcitabine in pancreatic cancer xenografts

**Fig. 8:**
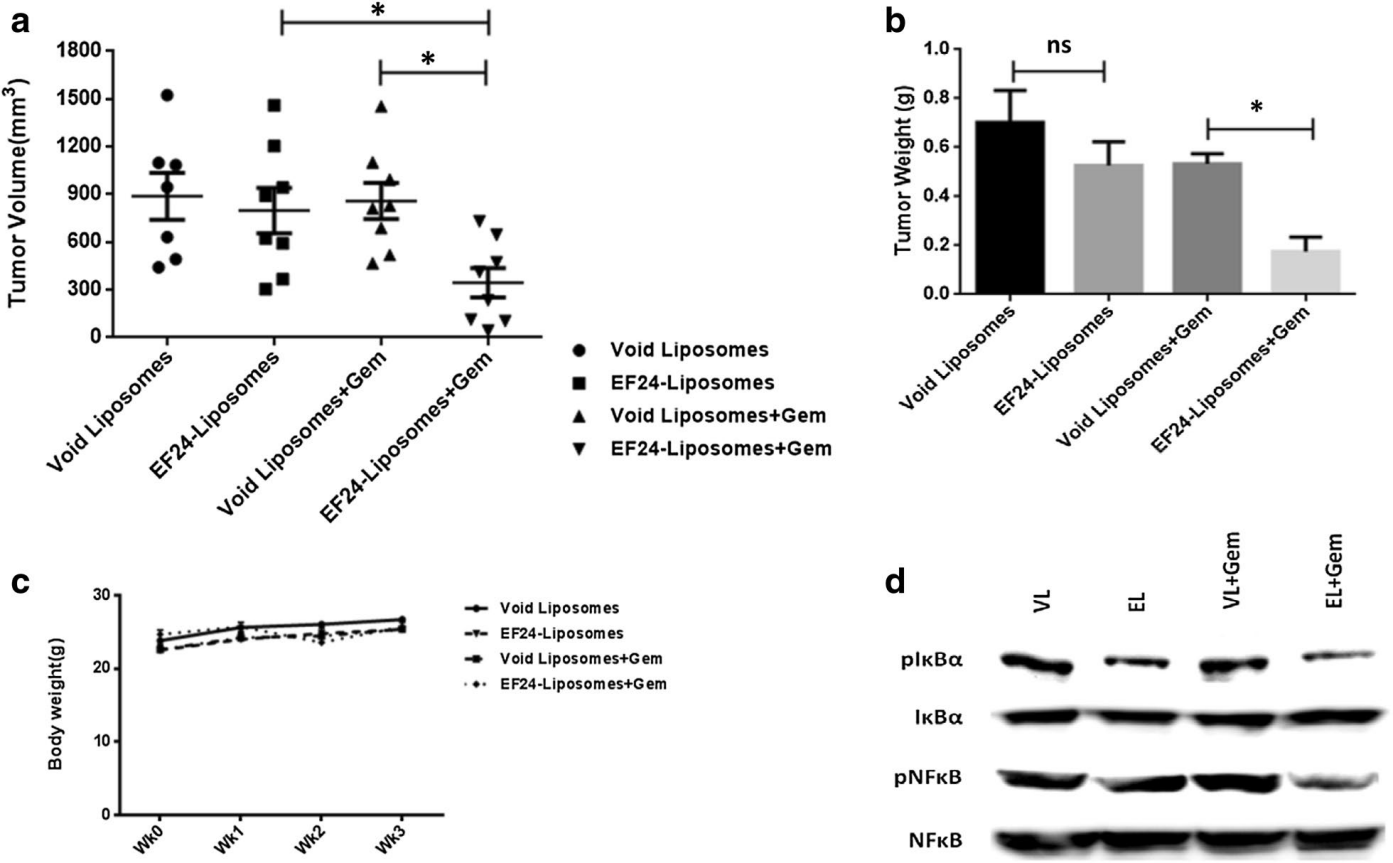
In vivo therapeutic effect of Lipo-EF24 on MIAPaCa xenograft tumor growth. **a** Growth of subcutaneous MIAPaCa xenografts was significantly inhibited in nude mice after treatment with Lipo-EF24 and gemcitabine in combination as shown by measured tumor volumes (n = 8) for each treatment arm after 3 weeks. Lipo-EF24 or gemcitabine used as single agents did not show any significant effect when compared to void liposomes as control. **b** Tumor weights measured at the end of treatment also showed significant reduction in the combination treatment arm as compared to either single agents or void liposomal control. **c** During the course of treatment, no difference in the mean body weight of mice was observed between the respective treatment arms. **d** Lipo-EF24 in combination with gemcitabine suppressed NF-kappaB activation in MIAPaCa xenografts, as demonstrated by marked inhibition of the phosphorylated form of its inhibitor protein I-kappa-B-alpha as well as NF-kappaB-p65, determined by western blot analysis of frozen tumor tissues

In vivo therapeutic efficacy of Lipo-EF24—administered as monotherapy as well as in combination with the standard-of-care cytostatic drug gemcitabine—was assessed using MIAPaCa pancreatic cancer xenografts. In line with previous reports gemcitabine caused significant growth retardation of MIAPaCa xenografts. As opposed to this, monotherapy with Lipo-EF24 lead to only marginal initial growth delay during the first 2 weeks of therapy (data not shown), but this effect did not persist during the following course of therapy, possibly due to in vivo selection of resistant subclones of neoplastic cells under monotherapy. This observation prompted investigation of a combinatorial regimen of Lipo-EF24 plus gemcitabine. Of note, significantly enhanced tumor growth inhibition was observed in mice that received a combination regimen of Lipo-EF24 plus gemcitabine as compared to single agent gemcitabine or Lipo-EF24 therapy, respectively (Fig. 8a). Likewise, significant decrease in the average final tumor weights was observed in the combination therapy as compared to the other three treatment arms (Fig. 8b). Thorough necropsy and histological examination of major organs was performed after the end of treatment and again did not reveal any apparent signs of toxicity or differences in mean body weights between the respective treatment arms (Fig. 8c). Xenograft tumor tissues were harvested at the end of treatment for analysis of drug target genes. Western blot analyses of excised tumors were found to be in line with previous in vitro results and showed decreased phosphorylation of I-kappa-B-alpha in both EF24 treatment arms (i.e. monotherapy or gemcitabine combination, respectively), while void liposomes or gemcitabine both did not show any such effect (Fig. 8d). Used as monotherapeutic agent, Lipo-EF24 failed to suppress NF-kappaB activation in vivo as observed in western blot analyses of resected xenograft tumor tissues. However, in combination with gemcitabine significant inhibition of phosphorylation of NF-kappaB-p65 and hence inactivation of NF-kappaB signaling could be detected even in the in vivo situation in resected tumor tissues.

## Discussion

Pancreatic cancer is a dismal disease and novel therapeutic options are urgently required. The plant alkaloid curcumin and active derivatives have recently been identified as a promising group of novel therapeutic agents in pancreatic as well as in others cancers [4, 5, 8]. For the parent compound curcumin, there is a large body of evidence documenting it’s in vitro activity against pancreatic cancer cells. These studies show that curcumin is a highly pleiotropic substance that acts on several oncogenic key signaling pathways, inluding inflammatory cytokines, JAK/STAT and NF-kappaB signaling pathways [29, 30]. Nano-encapsulation of curcumin has been shown to enable systemic in vivo administration of curcumin while preserving its antineoplastic on-target efficacy by our own group as well as by others [4, 5, 31–33], and early clinical evaluation of this concept is currently ongoing.

3,5-bis(2-fluorobenzylidene)-4-piperidone (EF24) was developed from curcumin as a synthetic monoketone compound that exhibited potent anticancer activity [8]. Tan et al. [34] found that EF24 upregulated cellular antioxidant responses as observed by the suppression of reactive oxygen species, generation and activation of antioxidant response element-dependent gene transcription in ovarian cancer cells and was more potent than curcumin in reducing VEGF secretion.

In hepatocellular carcinoma cells EF24 led to induction of apoptosis, inhibition of proliferation and G2/M cell cycle arrest that were accompanied by blockade of NF-kappaB signaling [25]. Moreover, intraperitoneal administration of EF24 solubilized using DMSO caused significant growth delay of PLC/PRF/5 or Hepa1-6 xenografts [25, 35]. While this study convincingly demonstrates the in vivo therapeutic potential of EF24 as putative future antineoplastic drug, solubilization in DMSO is not a viable concept that could be easily translated to clinical application in humans due to toxic side effects of concentrated DMSO. Therefore, novel ways of encapsulating EF24 will be required in order to evaluate its therapeutic efficacy in humans, and pegylated liposomal nanoparticles have already been proven to be a viable option for other agents [9, 10].

Adams et al. [23] suggested that EF24 induces cell-cycle arrest in G2/M and redox-mediated induction of apoptosis by altering mitochondrial function. In another study EF24 was found to inhibit proliferation of cisplatin-resistant ovarian cancer cells by induction of G2/M cell cycle arrest and to induce apoptosis by upregulation of membranous FasL and dephosporylation of Akt. In this study, EF24 treatment caused phosphorylation and upregulation of PTEN, and siRNA-mediated PTEN-knockdown partially rescued induction of apoptosis and cell cycle arrest [36]. In line with these previous observations by others, this present study shows that in pancreatic cancer cells treatment with EF24 leads to inhibition of proliferation and net cell growth as well as induction of apoptosis.

Moreover, EF24 has previously been identified as potent inhibitor of the oncogenic NF-kappaB signaling pathway that directly inhibits the catalytic activity of IkappaB kinase (IKK), blocks nuclear translocation of NF-kappaB and inhibits tumor necrosis factor (TNF)-alpha-induced IkappaB phosphorylation and degradation [24]. In this study the authors report that EF24 inhibited in vitro cell viability of various cancer cell lines (derived from lung, breast, ovarian and cervical cancer) with ten times higher potency as compared to curcumin. Confirming and extending these previous observations by others our data presented here show that in pancreatic cancer cells EF24 causes potent inhibition of NF-kappaB signaling.

Agashe et al. [37] described preparation of a liposomal nanoformulation of EF24 that differed from the approach chosen in this current study by first generating inclusion complexes of EF24 in hydroxypropyl-beta-cyclodextrin (HPβCD), which were then encapsulated into liposomes (designated “drug-in-CD-in liposome” approach). The liposomal EF24 particles were characterized in vitro and were found to inhibit proliferation of H441 lung adenocarcinoma as well as PC-3 prostate cancer cells more potently than non-encapsulated EF24 at a concentration of 10 µM. However, in this study in vivo evaluation of the synthesized EF24 nanoformulation was limited to pharmacokinetics and biodistribution studies in rats, and in vivo efficacy studies using suitable animal models of cancer were not carried out.

To the best of our knowledge our current study presented here is the first report of generation and physico-chemical characterization of a pegylated liposomal nanoformulation of EF24 (Lipo-EF24) for systemic administration that includes demonstration of in vivo antitumor efficacy in a murine xenograft model of human pancreatic cancer. In line with previous reports cited above, Lipo-EF24 led to direct induction of apoptosis as demonstrated by means of cleaved PARP and caspase-3 activity and inhibition of NF-kappaB signaling in pancreatic cancer cells. Of note, these molecular mechanisms could also be reproduced in vivo by analyzing Lipo-EF24-treated xenograft tumor tissues. Another remarkable finding of our study was the apparent non-toxicity of the Lipo-EF24 formulation presented here in murine in vivo models, which prompts the possibility of studying combination regimens. In fact, in xenograft experiments a convincing tumor growth inhibition was not achieved by Lipo-EF24 monotherapy but rather by its combination with the chemotherapeutic drug gemcitabine.

This observation seems to be in line with another recent report convincingly demonstrating that EF24 acts as strong inhibitor of FANCD2 monoubiquitylation (FANCD2-Ub) and targets the Fanconi Anemia (FA) pathway through inhibition of the NF-kappaB pathway kinase IKK [38]. Based on these observations one would infer from other cancer entities that there might be therapeutic synergism with other agents that put additional stress on DNA repair pathways, and in fact the authors found ATM-deficient tumor cells to be twofold more sensitive to EF24 treatment than matched wild-type controls; moreover, it was observed that EF24 specifically sensitized FA-competent cells to mitomycin C, a DNA crosslinker previously described to be particularly active in cancer cells with defective DNA repair pathways, including molecularly defined subsets of pancreatic cancer [39]. Based on these promising results it is highly tempting to speculate, whether there might also be synergism with other DNA-damaging agents such as cisplatin or irinotecan or with PARP inhibitors such as olaparib, that have also been found to be active in these subsets of cancers. Finally, a recent report suggested that gastrointestinal cancers including pancreatic cancers that are deficient in DNA mismatch-repair might be particularly vulnerable to therapeutic immune checkpoint inhibition by means of PD-1 antibodies [40], and it is therefore tempting to speculate whether treatment with Lipo-EF24 might likewise sensitize pancreatic cancer cells to this currently highly promising novel therapeutic strategy. Future studies examining these approaches therefore seem to be of obvious interest.

A recent report on preclinical hepatocellular carcinoma models suggested that EF24 might be used to overcome sorafenib-induced intratumoral hypoxia and therapy-resistance by inhibition of hypoxia-inducible factor (HIF)-1alpha by cytoplasmic sequestration and enhanced degradation [41]. Tumor hypoxia is a feature commonly observed in pancreatic cancer and is believed to be one of the key factors contributing to the general resistance of pancreatic cancer against therapeutic intervention. Of interest, another molecularly targeted approach to overcome hypoxia-mediated resistance against therapeutic intervention in pancreatic cancer has recently been suggested by inhibition of aberrant Hedgehog signaling, and in fact pharmacological Hedgehog inhibition was characterized by marked therapeutic synergism with administration of gemcitabine in vivo [19, 42], similarly to what has been found for Lipo-EF24 in this current study.

Another novel therapeutic approach that is currently being evaluated in pancreatic cancer is to block pro-inflammatory signaling pathways by means of small molecule inhibitors, promising phase 2 clinical data has recently been presented for the Janus Kinase inhibitor ruxolitinib [43, 44], although subsequent phase 3 data have been sobering. Since EF24 likewise has been found to inhibit JAK/STAT signaling and secretion of proinflammatory cytokines as well as other inflammatory pathways such as NF-kappaB and due to its apparent non-toxicity, it can be hypothesized that there might be therapeutic synergism when combining ruxolitinib therapy with Lipo-EF24, and this concept should be addressed in subsequent future studies.

Of note, it might be envisioned to co-encapsulate several compounds of such combination regimens (e.g. EF24 + gemcitabine + targeted agent) in one liposomal nanoparticle in an attempt to further enhance co-delivery to neoplastic tissues and thus increase therapeutic response. Future studies following up on this idea are currently already under way in our laboratory.

## Conclusions

Taken together the experimental results presented here demonstrate that liposomal formulation of EF24 shows therapeutic efficacy against pancreatic cancer tumors by suppression of NF-kappaB activation, mainly by inhibiting phosphorylation and subsequent degradation of its inhibitor protein I-kappa-B-alpha. Moreover, due to its very favorable toxicity profile Lipo-EF24 is also a promising candidate to further evaluate in combinatorial regimens together with gemcitabine or other targeted agents as pointed out above. The clinical value of such combinatorial approaches should be evaluated in future follow-up studies.

## Additional files

10.1186/s12951-016-0209-6 Additional information.

## Abbreviations

AUC: area under the concentration–time curve
BSA: bovine serum albumin
CFSE: carboxyfluorescein succinimidyl ester
DLS: dynamic light scattering
DMEM: Dulbecco’s Modified Eagles Medium
FELASA: Federation of European Laboratory Animal Science Associations
GC-MS: gas chromatography–mass spectrometry
HPβCD: hydroxypropyl-beta-cyclodextrin
LC–MS: liquid chromatography–mass spectrometry
MTS: 3-(4,5-dimethyl-2-yl)-5-(3-carboxymethoxyphenyl)-2-(4-sulfophenyl)-2H- tetrazolium
PBS: phosphate buffered saline
PEG-DSPE: 1,2-distearoyl-sn-glycero-3-phosphoethanolamine-*N*-[poly(ethylene glycol)- 2000]
POPC: 1-palmitoyl-2-oleoyl-sn-glycero-3-phosphocholine
RBC: red blood cell
RIPA: radioimmunoprecipitation assay
TEM: transmission electron microscopy
