# Supplementary material for: A liposomal formulation of the synthetic curcumin analog EF24 (Lipo-EF24) inhibits pancreatic cancer progression: towards future combination therapies
Source: J Nanobiotechnology. 2016 Jul 11;14:57. doi: 10.1186/s12951-016-0209-6 (PMC4940769; doi:10.1186/s12951-016-0209-6)
Supplement: Supplementary file 1 — 10.1186/s12951-016-0209-6 Additional information. [file 12951_2016_209_MOESM1_ESM.docx]

**Supplementary Information**

**Selection of an internal standard for LC-MS/MS measurements**

Since no isotopomer of EF-24 is commercially available, a structurally different molecule must be used as internal standard (ISTD). Reid et al. used a bromine analogue of EF-24, which also was not commercially available but was synthesized for their study (Reid et al. 2014). However, due to the lack of synthesis capacities and the future availability of the ISTD, a commercial alternative was sought. After literature search, three possible candidates were selected (supplementary figure S1). In order to select the most sensible candidate, a rough estimation of their relevant properties, above all the energy level of their highest occupied molecular orbitals (HOMOs) was performed *via* quantum-mechanical calculations. Table S1 gives a summary of the results. Clearly, besides the ISTD of Reid et al. also candidates 1 and 3 possess HOMO and LUMO energies similar to EF-24. As of these two, only candidate 1 and not candidate 3 also shows a basic nitrogen atom capable of being protonated under MS conditions, candidate 1 (InterBioScreen, Russia) was selected as ISTD.


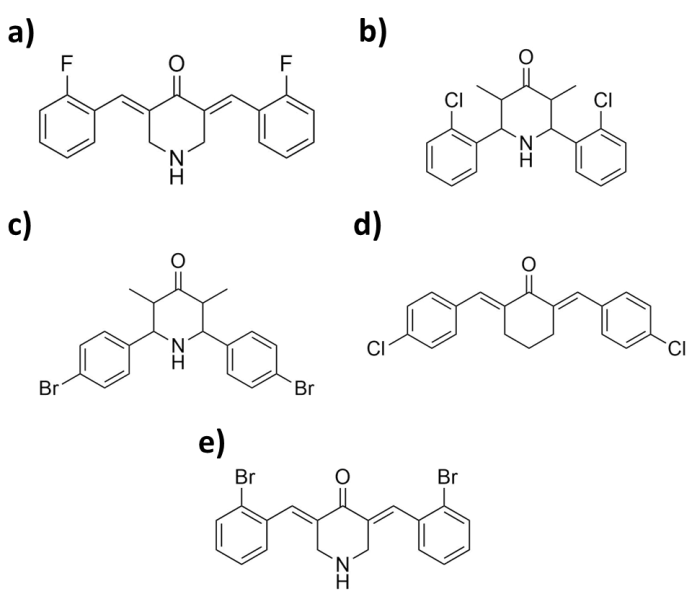


Figure S1: Structural formulae of (a) EF-24, (b) Candidate 1, (c) Candidate 2, (d) Candidate 3 and (e) the ISTD chosen by Reid et al.

Table S1: Results of quantum-chemical calculations. Note that EF-24, Candidate 3 and the ISTD of Reid et al. were calculated in the (E,E)-configuration.

| Molecule | RI-BP86/SV(P) | | RI-BP86/TZVP | |
| --- | --- | --- | --- | --- |
|  | HOMO / eV | LUMO / eV | HOMO / eV | LUMO / eV |
| EF-24 | -5.351 | -3.177 | -5.612 | -3.319 |
| Candidate 1 | -5.390 | -1.929 | -5.650 | -2.146 |
| Candidate 2 | -5.488 | -2.026 | -5.772 | -2.241 |
| Candidate 3 | -5.541 | -3.194 | -5.694 | -3.340 |
| ISTD of Reid et al. | -5.272 | -3.134 | -5.661 | -3.351 |

**LC-MS/MS analyses**

LC-MS/MS analyses were carried out on a Waters Xevo TQ-S Triple-quad system equipped with a Waters Acquity I-Class UPLC system, an FTN sample manager, binary solvent manager and TUV detector. A Waters Acquity UPLC BEH C18 column (130 Å, 1.7 µm, 2.1 mm × 100 mm) together with a Waters Acquity UPLC BEH C18 VanGuard pre-column (130 Å, 1.7 µm, 2.1 mm × 5 mm) was used for separation. Data recording was achieved with the MassLynx 4.1 software package, data processing and quantification was performed with the integrated TargetLynx software using a quadratic regression model. Calibration curves showed R²>0.99 for all measurements. Mobile phase A was a solution of 0.1 % formic acid in H_2_O, mobile phase B a solution of 0.1 % formic acid in methanol (all in LCMS grade). The elution program was as follows: Initial mixture of 60 % A (0 – 10 min), linear gradient towards 20 % A (10 – 15 min), hold at 20 % A (15 – 18 min), linear gradient towards 60 % A (18 – 20 min), hold at 60 % A (20 – 22 min). 10 µL of the respective sample was injected at 0 min; the flow rate was 0.2 mL/min throughout the entire acquisition. The column temperature was set to 45 °C while the sample manager temperature was set to 10 °C. The TUV detector was bypassed during the acquisition. MRM transitions of EF-24 and the internal standard were recorded in ESI^+^ mode throughout the entire acquisition. Transitions of EF-24 were: 312.2 > 149.0 (collision energy: 26 V; role: quantifier) and 312.2 > 109.7 (CE: 20 V; qualifier); transitions of the internal standard were: 348.1 > 140.0 (CE: 26 V; quantifier) and 348.1 > 125.0 m/z (CE: 26 V, qualifier). Minor transitions of EF-24 could also be observed at 121 and 292 m/z, respectively. A possible fragmentation scheme based on these observations has been shown in supplementary figure S2. Argon was used as collision gas. Source voltage was 2.8 kV, cone voltage 20 V, desolvation temperature 200 °C, source temperature 150 °C, source flow 150 L∙h^–1^ and desolvation gas flow 350 L∙h^–1^. Concentration of the internal standard was 20 ng/mL in all samples and calibration standards. Beside a double blank and blank sample, six calibration standards were mixed with concentrations of EF-24 of 40, 80, 150, 400, 800 and 1190 ng/mL.

**Quantum-chemical calculations(QCCs)**

QCCs were performed with the ORCA 3.0.3 software package (Neese 2012). Molecular input structures were created with the help of the Avogadro 1.1.1 software package (Hanwell et al. 2012) equipped with an input file generator tool for ORCA. Pre-optimization was achieved with the MM2 energy optimization algorithm implemented in Avogadro. Structure optimization calculations including orbital calculations were then performed with ORCA on an RI-BP86/SV(P) (grid size 4, default convergence criteria) level of theory (Weigend und Häser 1997) (Weigend et al. 1998) (Becke 1988) (Perdew 1986) (Dirac, P. A. M. 1929) (Slater 1951) (Vosko et al. 1980) (Schäfer et al. 1992) (Schäfer et al. 1994) (Weigend und Ahlrichs 2005). Subsequent frequency calculation was used to confirm that a true minimum on the energy hypersurface was found by checking the frequencies for absence of imaginary modes. The optimized coordinates were then used for an additional structure optimization on the RI-BP-86/TZVP level of theory. Visualization of the results was done with the Avogadro software package .


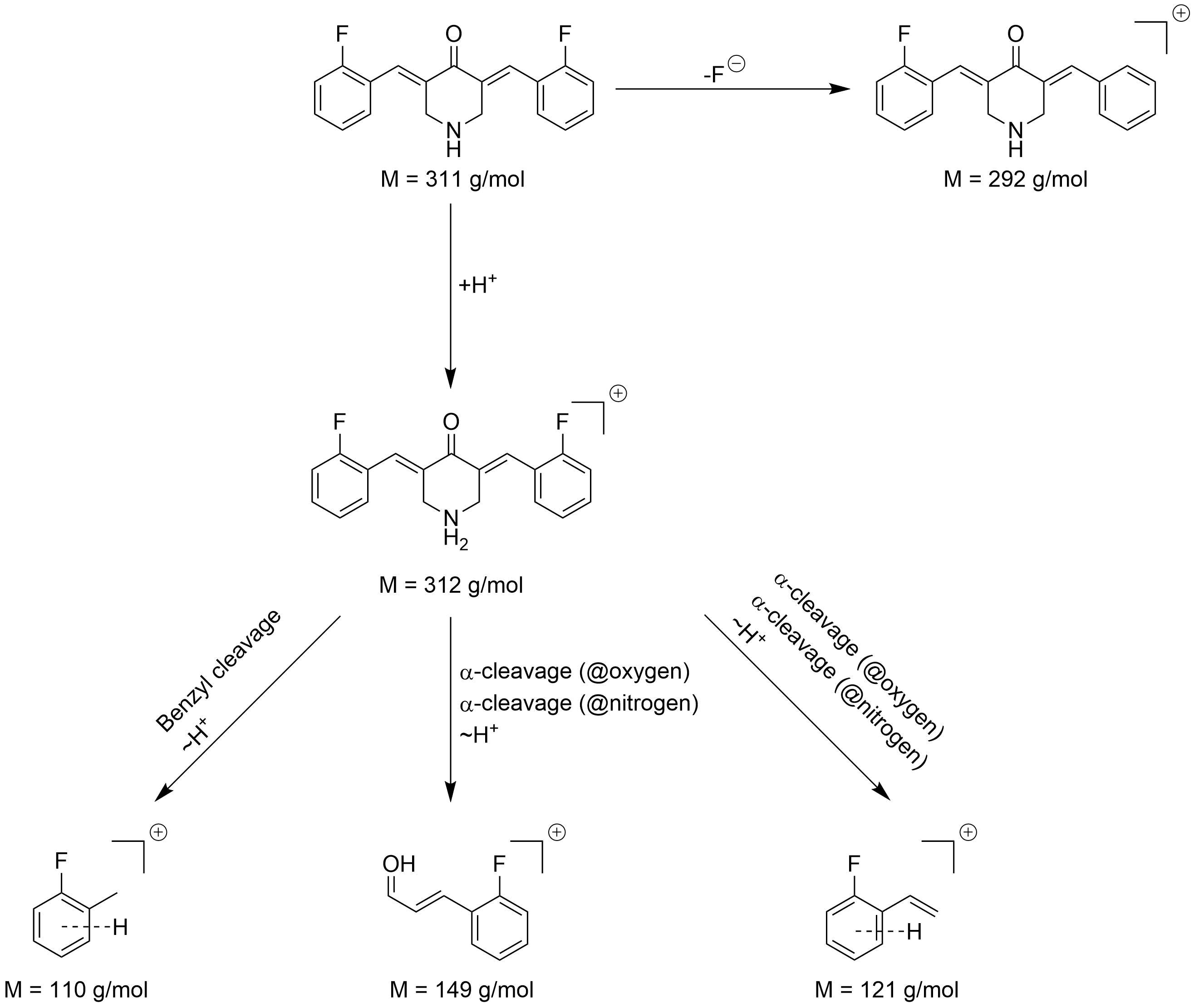


Figure S2: Possible fragmentation patterns of EF-24 according to the most intensive MS peaks.

References

Becke, A. D. (1988): Density-functional exchange-energy approximation with correct asymptotic behavior. In: *Phys. Rev. A* 38 (6), S. 3098–3100. DOI: 10.1103/PhysRevA.38.3098.

Dirac, P. A. M. (1929): Quantum Mechanics of Many-Electron Systems. In: *Proceedings of the Royal Society A: Mathematical, Physical and Engineering Sciences* 123 (792), S. 714–733. DOI: 10.1098/rspa.1929.0094.

Hanwell, Marcus D.; Curtis, Donald E.; Lonie, David C.; Vandermeersch, Tim; Zurek, Eva; Hutchison, Geoffrey R. (2012): Avogadro: an advanced semantic chemical editor, visualization, and analysis platform. In: *J Cheminf* 4 (1), S. 17. DOI: 10.1186/1758-2946-4-17.

Neese, Frank (2012): The ORCA program system. In: *WIREs Comput Mol Sci* 2 (1), S. 73–78. DOI: 10.1002/wcms.81.

Perdew, John P. (1986): Density-functional approximation for the correlation energy of the inhomogeneous electron gas. In: *Phys. Rev. B* 33 (12), S. 8822–8824. DOI: 10.1103/PhysRevB.33.8822.

Reid, Joel M.; Buhrow, Sarah A.; Gilbert, Judith A.; Jia, Lee; Shoji, Mamoru; Snyder, James P.; Ames, Matthew M. (2014): Mouse pharmacokinetics and metabolism of the curcumin analog, 4-piperidinone,3,5-bis[(2-fluorophenyl)methylene]-acetate(3E,5E) (EF-24; NSC 716993). In: *Cancer Chemotherapy and Pharmacology* 73 (6), S. 1137–1146. DOI: 10.1007/s00280-014-2447-3.

Schäfer, Ansgar; Horn, Hans; Ahlrichs, Reinhart (1992): Fully optimized contracted Gaussian basis sets for atoms Li to Kr. In: *J. Chem. Phys.* 97 (4), S. 2571. DOI: 10.1063/1.463096.

Schäfer, Ansgar; Huber, Christian; Ahlrichs, Reinhart (1994): Fully optimized contracted Gaussian basis sets of triple zeta valence quality for atoms Li to Kr. In: *J. Chem. Phys.* 100 (8), S. 5829. DOI: 10.1063/1.467146.

Slater, J. C. (1951): A Simplification of the Hartree-Fock Method. In: *Phys. Rev.* 81 (3), S. 385–390. DOI: 10.1103/PhysRev.81.385.

Vosko, S. H.; Wilk, L.; Nusair, M. (1980): Accurate spin-dependent electron liquid correlation energies for local spin density calculations: a critical analysis. In: *Can. J. Phys.* 58 (8), S. 1200–1211. DOI: 10.1139/p80-159.

Weigend, Florian; Ahlrichs, Reinhart (2005): Balanced basis sets of split valence, triple zeta valence and quadruple zeta valence quality for H to Rn: Design and assessment of accuracy. In: *Phys. Chem. Chem. Phys.* 7 (18), S. 3297. DOI: 10.1039/B508541A.

Weigend, Florian; Häser, Marco (1997): RI-MP2: first derivatives and global consistency. In: *Theoretical Chemistry Accounts: Theory, Computation, and Modeling (Theoretica Chimica Acta)* 97 (1-4), S. 331–340. DOI: 10.1007/s002140050269.

Weigend, Florian; Häser, Marco; Patzelt, Holger; Ahlrichs, Reinhart (1998): RI-MP2: optimized auxiliary basis sets and demonstration of efficiency. In: *Chemical Physics Letters* 294 (1-3), S. 143–152. DOI: 10.1016/S0009-2614(98)00862-8.
